# Nutritional Strategies to Prevent Muscle Loss and Sarcopenia in Chronic Kidney Disease: What Do We Currently Know?

**DOI:** 10.3390/nu15143107

**Published:** 2023-07-11

**Authors:** Giulia Massini, Lara Caldiroli, Paolo Molinari, Francesca Maria Ida Carminati, Giuseppe Castellano, Simone Vettoretti

**Affiliations:** 1 Department of Clinical Sciences and Community Health, University of Milan, 20122 Milan, Italy; giulia.massini@unimi.it (G.M.); giuseppe.castellano@unimi.it (G.C.); 2 Unit of Nephrology, Dialysis and Kidney Transplantation, Fondazione IRCCS Ca’ Granda Ospedale Maggiore Policlinico di Milano, 20122 Milan, Italy; lara.caldiroli@policlinico.mi.it (L.C.); paolo.molinari1@unimi.it (P.M.); ida.carminati@gmail.com (F.M.I.C.)

**Keywords:** chronic kidney disease, sarcopenia, malnutrition, muscle loss, diet, protein intake

## Abstract

Loss of muscle mass is an extremely frequent complication in patients with chronic kidney disease (CKD). The etiology of muscle loss in CKD is multifactorial and may depend on kidney disease itself, dialysis, the typical chronic low-grade inflammation present in patients with chronic kidney disease, but also metabolic acidosis, insulin resistance, vitamin D deficiency, hormonal imbalances, amino acid loss during dialysis, and reduced dietary intake. All these conditions together increase protein degradation, decrease protein synthesis, and lead to negative protein balance. Aging further exacerbates sarcopenia in CKD patients. Nutritional therapy, such as protein restriction, aims to manage uremic toxins and slow down the progression of CKD. Low-protein diets (LPDs) and very low-protein diets (VLPDs) supplemented with amino acids or ketoacids are commonly prescribed. Energy intake is crucial, with a higher intake associated with maintaining a neutral or positive nitrogen balance. Adequate nutritional and dietary support are fundamental in preventing nutritional inadequacies and, consequently, muscle wasting, which can occur in CKD patients. This review explores the causes of muscle loss in CKD and how it can be influenced by nutritional strategies aimed at improving muscle mass and muscle strength.

## 1. Introduction

Loss of muscle mass is an extremely frequent complication in patients with chronic kidney disease (CKD) [1,2,3]. This results from increased protein catabolism and reduced protein synthesis, with a negative protein balance [4]. Protein-energy wasting (PEW), malnutrition, sarcopenia, and cachexia denote nutritional derangements that often characterize CKD patients. These conditions, while having distinct definitions, share criteria and have common clinical outcomes, including malnutrition, characterized by loss of body weight, muscle mass, and body fat due to low energy and nutrient intake; PEW differs from malnutrition for the low-grade inflammation as an additional etiological condition [5]. Sarcopenia is defined as the loss of muscle mass and muscle strength that occurs with aging, while cachexia is a syndrome characterized by chronic inflammation and increased catabolism of muscle proteins, with severe muscle loss that may or may not be accompanied by loss of body fat [6].

Since the European Working Group for Sarcopenia for Older People (EWGSOP) [6] published the first consensus, several studies have been published about sarcopenia in CKD patients.

The aim of this review is to explore the etiology of loss of muscle mass in CKD, and how it can be affected by nutritional strategies aiming to improve muscle mass and muscle strength.

## 2. Cause of Muscle Loss and Sarcopenia in CKD Patients

**Figure 1:**
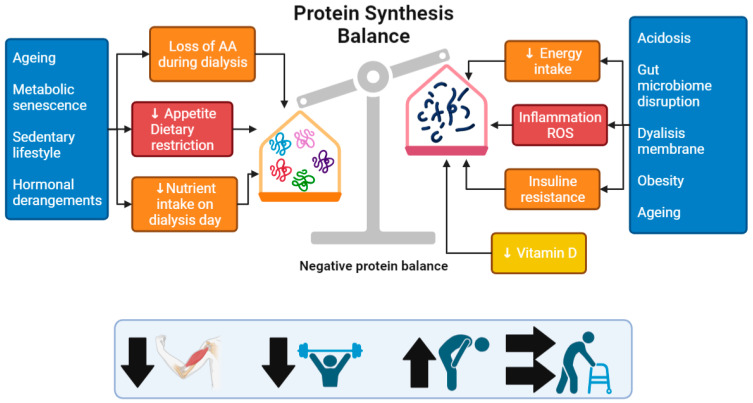
Etiologic factors leading to muscle loss in chronic kidney disease.

The etiology of muscle loss in CKD is multifactorial and may depend on the severity of the kidney disease, dialysis, and the typical chronic low-grade inflammation of CKD patients. All these conditions together increase protein degradation, decrease protein synthesis, and lead to negative protein balance [1,7,8] (Figure 1). Other factors related to CKD that promote this vicious cycle include the development of metabolic acidosis, insulin resistance, and vitamin D deficiency [1,9,10,11,12,13,14]. Metabolic acidosis stimulates protein degradation through two systems, caspase-3 and the ubiquitin–proteasome system (UPS) [1,15] and through the promotion of insulin and growth hormone (GH) resistance [16]. Mild metabolic acidosis reduces insulin binding to its receptor, whereas plasma membrane insulin receptor expression is unaffected by extracellular pH [17,18,19,20]. In addition, insulin receptor phosphorylation, and thus activation, is significantly reduced at low pH. The level of phosphorylation of Akt, which is a downstream target of the insulin signaling pathway, is also reduced at low pH [18]. In addition, low pH decreases the expression of adiponectin [17,21], which enhances insulin sensitivity in skeletal muscle [17,22,23].

Vitamin D deficiency, on the other hand, leads to a reduction in insulin secretion [1,24,25] and to a decrease in the stimulus for protein synthesis in the muscle caused by a reduction in vitamin D receptors and a reduction in calcium influx [14]. In addition, a negative protein–energy balance can be caused by other factors such as hormonal imbalances (testosterone, insulin growth factor (IGF-1), and GH resistance), substantial loss of amino acids during the dialysis procedure [1,26], and reduced dietary energy and protein intake, which has been shown to be lower on the day of dialysis [1,27]. Other factors contributing to CKD-related inflammation are pro-inflammatory response caused by dialysis membranes bio-incompatibility [4], intestinal dysbiosis, and mucosal barrier impairment [28,29,30]. The latter can result from uremia and reduced fiber intake. The second mentioned is necessary to lower potassium intake. Fiber reduction leads to an increase in protein fermentation and its metabolites (ammonium, thiols, phenols, and indoles) that accumulate in CKD patients due to reduced renal clearance [1,28]. Moreover, intestinal dysbiosis in uremia conditions can lead to increased endotoxins levels which induce inflammatory cascades and maintain low-grade systemic inflammation. Obesity in patients with chronic renal failure can also lead to an increase in inflammatory status due to adipocyte dysfunction, characterized by an increased synthesis of cytokine and adipokines [31]. Lastly, a very important but underestimated cause of muscle loss and sarcopenia in CKD patients is low physical activity [1,32].

All of these conditions together result in a negative protein balance that can lead to muscle loss, poor muscle strength, low physical performance, disability, and frailty [33] (Figure 1). In addition, aging is another major cause of sarcopenia in end-stage kidney disease (ESKD); elderly CKD patients appear to be more vulnerable to muscle degradation compared to the younger CKD population. This was demonstrated by Çelik et al. [1,34], who noticed that hemodialysis (HD) patients aged 65 years and older had a lower fat-free mass (FFM) index, creatinine, and dry body weight than younger patients (<65 years). D’Alessandro et al. [1,35] observed in CKD stage 3a-4 patients that sarcopenia was more frequent in older older adults (>75 years) than younger older adults (65–74 years), with a prevalence of 55% vs. 12.5%, respectively. In particular, this study showed that all three components of sarcopenia–skeletal muscle index, hand grip strength (HGS), and physical performance tests were significantly lower in elderly patients with CKD [35]. A study based on the EQUAL study, which evaluated 1334 patients, showed that the risk of protein-energy wasting (PEW) in patients with an eGFR < 20 mL/min increased with age and was characterized by low muscle trophism [36]. Hence, aging appears to contribute to sarcopenia development in CKD.

## 3. Malnutrition and Sarcopenia in Patients with Low-Protein Nutritional Therapy

Protein restriction in non-dialyzed patients offers various benefits, such as reducing the build-up of toxic intermediates in the blood and tissues, called “uremic toxins” [37]. In fact, in CKD patients, the elimination of nitrogen waste products from protein and amino acid catabolism gradually decreases, and the accumulation of these toxins leads to anorexia and nausea, resulting in a further decrease in nutrient intake. This can cause a gradual depletion of the body’s protein and energy stores [38]. In addition to managing uremic toxicity, nutritional treatment for CKD involves careful monitoring of phosphate, sodium, protein quality, and energy intake. This is because other important goals include slowing the progression of renal failure and proteinuria, achieving better control of blood pressure, and fully correcting metabolic acidosis. The recommended low-protein diet (LPD) for these patients typically contains 0.55–0.80 g of protein/kg/day, with over 50% of the protein being of high biological value [39,40].

Patients in advanced CKD stages (4–5) are more commonly prescribed very low-protein diets (VLPDs) containing 0.3–0.4 g of protein/kg/day. These VLPDs are often supplemented with amino acids (AAs) and/or ketoacids (KA) in the form of a 7–15 g mixture of KA or hydroxyacid analogs of five essential AAs (plus tryptophan, histidine, threonine, and lysine) [39,40]. Therefore, nutritional treatment for CKD patients requires a high energy intake as a necessary component. Studies have shown that there is a direct relationship between dietary energy intake and nitrogen balance in clinically stable CKD patients who consume low-protein diets (0.55 to 0.60 g/kg/day grams of protein per kilogram of body weight per day). It has been found that an energy intake providing approximately 35 kcal/kg/day is more likely to maintain a neutral or positive nitrogen balance. Therefore, ensuring an adequate energy supply is crucial in the nutritional management of CKD patients [37,41].

The shift toward an elderly and often frail population with CKD and ESKD requires a thorough assessment of body composition and muscle function in order to calculate patients’ protein requirements more precisely and formulate a balanced diet. This is because protein needs may differ in elderly patients due to changes in body composition, reduced kidney function, and other age-related factors [42]. Elderly individuals often have a reduced appetite, consume smaller meals, and have lower energy intake, which can lead to weight loss. Weight loss in elderly subjects is associated with increased morbidity and mortality due to microinflammation and excess cytokine production. In addition, decreased insulin sensitivity in elderly subjects is linked to frailty and cognitive impairment [43]. The traditional recommendation for protein intake of 0.8 g of protein/kg/day for adults of all ages is currently being debated for elderly subjects. In the prescription of daily protein intake, it is important to consider that protein restriction in elderly individuals with CKD may potentially cause or exacerbate sarcopenia. According to the EWGSOP2 definition, sarcopenia is characterized by progressive and generalized loss of muscle mass and strength that can occur not only in older age but also earlier in life, with increased risk of adverse outcomes including physical disability, poor quality of life, and death [1]. So, what is the better protein intake for an elder CKD patient? Studies suggest that elder individuals may need higher amounts of protein (1.0–1.2 g of protein/kg/day) to preserve lean body mass [44]. In particular, ESPEN guidelines recommend a minimum protein intake of 0.8 g of protein/kg/day when the estimated glomerular filtration rate (eGFR) is less than 30 mL/min [43].

For several decades, a low-protein diet has been proposed as a solution, but the balance between the benefits and harms of this dietary regimen has not been totally resolved.

Several studies in patients with CKD (stage 3–5, not on dialysis) have evaluated the use of controlled protein in elderly patients. They have shown that, with an adequate energy intake, a good nutritional status can be maintained even in these patients [1].

Connie M. Rhee et al., in 2018, aimed to conduct a systematic review and meta-analysis examining the effect of LPD on the clinical management of uremia and its complications in patients with CKD. The authors identified 16 controlled trials of a low-protein diet in CKD. Studies were divided into 3 groups: those comparing LPD (0.4–0.8 g/kg/day) with higher-protein diets (>0.8 g/kg/day) and those comparing VLPD (<0.4 g/kg/day) with LPD. In the first group evaluation, a reduction in the risk of progression to ESRD, an increase in serum bicarbonate at 1 year, and serum phosphorus comparable at 1 year were observed in LPD patients. The second group analysis highlighted a reduction in the risk of the progression to ESRD, an increase in GFR value at 1 year, and a decrease in serum urea at 1 year in VLPD. This meta-analysis concluded that a low-protein diet represents valid conservative management in non-dialysis-dependent CKD patients. This could be considered a potential option for CKD patients to avoid or defer dialysis initiation and slow down the progression of CKD. According to this study, the risk of PEW and cachexia remains minimal [45]. In accordance with the previous study, a Cochrane systematic review conducted in 2021 highlighted that very low-protein diets (0.3 to 0.4 g/kg/day with supplements of essential amino acids and keto-analogs) compared to low or normal protein intakes, can probably reduce the progression to ESKD (moderate certainty evidence). In participants with CKD stage 3, compared to normal protein intake (0.8 to ≥1.0 g/kg/day), a low-protein diet (0.5 to 0.6 g/kg/day) does not appear to influence the number of patients that progress to ESKD (low certainty evidence). However, limited data were available on adverse effects, in particular, weight differences and protein energy malnutrition [46].

How muscle protein metabolism adapts to a low-protein intake in humans is still an open question. An interesting study was conducted by Garibotto et al. 2018; the authors addressed this issue by measuring muscle protein turnover in 2 cohorts of patients with CKD through 2 levels of dietary protein: LPD providing 0.55 g protein/kg per day (*n* = 11) and VLPD (*n* = 6) providing 0.45 g protein/kg per day, supplemented (0.1 g/kg) with EAA and KA. This study showed that in patients with CKD, the skeletal muscle responds to an LPD through a shift in muscle protein dynamics that occurs when protein intake is reduced. This change is characterized by a decrease in protein degradation, no significant change in protein synthesis, and an overall improvement in the efficiency of protein metabolism. These changes help minimize muscle nitrogen (N) losses and can contribute to the preservation of nitrogen balance and muscle mass when a decrease in nitrogen intake occurs. The study highlighted how, compared to a diet containing 1.1 g/kg of protein, a low-protein diet (LPD) with a protein content of 0.55 g/kg resulted in the following effects: a 17% to 40% reduction in muscle protein degradation and net protein balance, respectively, no significant change in muscle protein synthesis, a slight decrease (approximately 7%, *p* < 0.06) in whole-body protein degradation, and an improvement in the efficiency of muscle protein turnover. In comparison to the LPD, a VLPD led to the following outcomes: no notable change in muscle protein degradation and an approximate 50% decrease in negative net protein balance, along with an improvement in the efficiency of muscle protein turnover [47].

## 4. Nutritional Strategies to Prevent or Improve Sarcopenia in Pre-Dialysis

Adequate nutritional and dietary support are fundamental in all phases of CKD. Nutritional therapy is essential in CKD patient health care because it plays an important role in slowing the progression to ESKD and delaying the onset of renal replacement therapy. However, the goal of dietetic intervention is also preserving good nutritional status and avoiding PEW, electrolyte imbalances, and bone and mineral abnormalities.

In 2001, Castaneda et al. conducted a randomized controlled trial to assess the effectiveness of resistance training in enhancing protein utilization and increasing muscle mass among patients with CKD on a low-protein diet (LPD) [48]. A total of 26 older patients with moderate renal insufficiency participated in the study. They were randomly divided into two groups; one group (*n* = 14) followed an LPD along with resistance training, while the other group (*n* = 12) only followed the LPD for a period of 12 weeks. In the group of patients who participated in resistance training, authors observed a significant increase in total body potassium and cross-sectional areas of type I and II muscle fibers compared to the group that did not engage in resistance training. Additionally, leucine oxidation and serum prealbumin levels showed significant improvements in the resistance training group. The patients who underwent resistance training also maintained their body weight, unlike the other group. Furthermore, the improvement in muscle strength was significantly greater in the resistance training group compared to the counterpart that did not engage in resistance training (*p* < 0.001).

In 2012, a randomized controlled trial was conducted to assess the efficacy of exercise and amino acid supplementation in improving muscle mass and strength in elderly Japanese sarcopenic women (*n* = 155) [49]. Participants were randomly assigned to one of four groups: exercise and amino acid supplementation (*n* = 38), exercise (*n* = 39), amino acid supplementation (*n* = 39), or health education (*n* = 39). The exercise group participated in a comprehensive 60-min training program that took place twice a week. On the other hand, the amino acid supplementation group consumed 3 g of a mixture rich in essential amino acids, particularly leucine, twice a day for a duration of 3 months. Results highlight that the combination of exercise and amino acid supplementation in women with sarcopenia may have a significant impact on improving not only muscle strength but also the combined variables of muscle mass and walking speed, as well as muscle mass and strength.

For a long time, the reduction in protein intake has been the main nutritional treatment for patients affected by chronic renal failure, but it is not the only aspect of dietary management to consider in CKD patients. Other features play an important role, such as changes in sodium, phosphorus, and energy intake, the source of protein (animal or plant-derived), lipids uptake, essential amino acid, and ketoacid supplementation in LPD/VLPD therapy [50]. In addition, the response of muscle metabolism to protein restriction in humans is a delicate issue, and the risk of sarcopenia in these patients is high. Therefore, an important question should be asked: “Could sarcopenia be prevented or improved through nutritional approach in CKD patients?”.

An improvement in nutrient intake in malnourished or sarcopenic patients could be achieved with energy and protein supplementation. It is important to note that protein supplementation alone without exercise may be of limited benefit; several studies suggest anabolic benefits when supplementation is given immediately after exercise due to synergistic effects [1,51]. In CKD patients with sarcopenia, increasing protein intake alone without exercise may have limited effect. The combination of an exercise program and an increase in protein intake is effective at improving muscle mass and muscle strength in sarcopenic CKD patients [52].

CKD is a medical condition in which the amino acid requirements of the individual are different from the normal state, and this condition needs to be taken into account when supplementing with protein. The metabolism of certain essential amino acids such as Leucine, Valine, Arginine, Tyrosine, Tryptophan, Cysteine, and others is affected, and this condition impacts muscle health and function. Changes in the metabolism of amino acids in the muscle that are specific to uremia may contribute to an increase in catabolic processes or a decrease in anabolic processes. Since arginine is the precursor of nitric oxide (NO) in uremia, the atypical supply of arginine to the tissues may have adverse effects. The reduced availability of NO is associated with hypertension, impaired endothelial cell function and integrity, accelerated atherosclerosis, progression of CKD, depression, and behavioral changes [53,54]. In addition, NO acts as a mediator for several hormones, such as insulin growth factor-I (IGF-I) and growth hormone (GH). Therefore, a decrease in NO availability could affect hormonal activity and muscle health [55]. Even tryptophan is an essential amino acid for protein synthesis. A reduction in its plasma levels could lead to an increased risk of cardiovascular disease and a reduction in melatonin synthesis [56]. Leucine is the most potent amino acid in stimulating muscle anabolism and inhibiting catabolism. A reduction in its concentration leads to a decrease in muscle function. The kidney plays a crucial role in the oxidation of the leucine ketoanalogue. However, low levels are found in the plasma and muscle cells of patients with CKD [57,58]. Finally, patients with CKD have an unusual pattern of sulfur-containing amino acids in their blood: free and protein-bound cysteine (especially homocysteine) are high in the plasma of uremic patients, while methionine is normal, and taurine is low. Part of the increase in the anion gap observed in patients with end-stage renal disease (ESRD) is due to the progressive accumulation of sulfate in the blood. This is caused by a reduction in urinary sulfate excretion [57,58]. These irregularities are mainly caused by reduced food intake, hyperinsulinemia, urinary protein losses, and increased calcium (Ca) metabolism of branched-chain amino acids in muscle. Several of the abnormalities observed in CKD may be explained by the loss of renal tissue responsible for metabolizing amino acids [57,58].

Considering the above, the supplementation of essential amino acids (EAA), branched-chain amino acids (BCAA), and keto acids (KAs), in combination with LPD, represents a useful nutritional strategy to improve uremic sarcopenia. This dietetic approach would allow maintaining protein restriction as a key factor in the conservative management of CKD, improving appetite, ensuring the correct AA and albumin concentration in plasma, and enhancing muscle strength. KAs are precursors of corresponding amino acids since they can undergo transamination. KAs can be utilized in place of their respective EAAs without providing nitrogen products while re-using available nitrogen already in excess during CKD. The administration of KAs has been proposed to improve protein status, limiting the nitrogen burden on the body. Different compositions of KAs and EAAs have been tested, most of them containing four KAs (of the EAA isoleucine, leucine, phenylalanine, and valine), one hydroxyacid (of the EAA methionine), and four amino acids considered essential in CKD (tryptophan, threonine, histidine, and tyrosine).

In addition, the calcium content of the KA preparation could allow the correction of mineral metabolism disorders. However, it could be a cause of hypercalcaemic episodes and unpleasant gastrointestinal symptoms, which are the main side effects of this supplement [59].

Several studies were conducted to prove LPD/VLPD + KAs safety. Barsotti et al., in 1981 [53], demonstrated that an LPD supplemented with KAs and amino acids allowed a slowing of the progression of renal disease and reduced CV risk. These results have been explained by the fact that the supplemented LPD carried a low amount of phosphorus which induced a negative balance to determine a decrease in serum phosphorus [44]. In 2016, Zemchenkov et al. evaluated the effect of an LPD (0.60 g protein/kg body weight/day) supplemented with ketoanalogue (EAA/KA) therapy on the rate of eGFR decline in 96 patients with CKD stages 3B-5. The study showed a reduction in CKD progression, particularly in older patients with higher time-averaged proteinuria (PU) and lower phosphate levels, particularly in women [60]. In 2018, Milovanova et al. conducted a prospective, randomized, controlled trial in 79 CKD 3b-4 patients to compare the possibilities of LPD + KA and isolated LPD on their impact on FGF-23, whose plasma changes in patients with advanced CKD are associated with bone mineral disorders, metabolic acidosis, oxidative stress, chronic inflammation, and protein-energy wasting. Patients were randomized into two groups: the LPD + KA (0.1 g/kg body weight/day) group (42 patients) and the control group (37 patients), which followed the LPD indications only (0.6 g/kg body weight/day, consisting of 0.3 g vegetable protein and 0.3 g animal protein, with total phosphorus content ≤800 mg/day and daily energy intake 34–35 kcal/kg/day). After 14 months, patients in group 2 (LPD alone) achieved a statistically significant reduction in BMI (*p* = 0.046), including muscle mass in men (*p* = 0.027) and women (*p* = 0.044), according to bioimpedance analysis; no signs of protein-energy malnutrition were observed in the first group (LPD + KA). In the control group, the authors found a statistically significant decrease in serum total protein (*p* = 0.039) and transferrin (*p* = 0.048) levels, while mean serum albumin levels remained within the normal range in both groups. Serum urea and plasma FGF-23 levels were lower in the LPD + KA patients at the end of the study. Serum bicarbonate levels were statistically higher in group 1 than in group 2 [47,61].

Neutral or positive nitrogen balance requires adequate energy intake, and low energy intake can lead directly to protein wasting, as discussed above. Energy supplements with controlled fat and carbohydrate content have been proposed in combination with LPD to reduce the risk of malnutrition, complicated by a reduction in muscle mass and, hence, sarcopenia [54]. A prospective, randomized, controlled clinical trial conducted in 2013 evaluated the effects of a non-protein calorie (NPC) supplement on renal function and nutritional status in patients on a low-protein diet. The study included 109 CKD patients with stage 3 to 4 disease, all of whom received individualized dietary advice to achieve a daily protein intake of 0.6 to 0.8 g and a daily energy intake of 30 to 35 kcal/kg. The intervention group (55 CKD patients) consumed 0.6 g protein, 8.2 g lipids, 30.9 g carbohydrates, and 1.9 g fiber with an energy content of 200 kcal. The control group (54 patients) received only dietary advice. The authors showed that dietary protein intake and urinary protein excretion decreased significantly in the intervention group and were significantly lower than in the control group. Furthermore, serum creatinine and urea nitrogen levels decreased significantly, and eGFR increased significantly compared to baseline [62].

The study showed that controlled energy supplementation would improve adherence to the same diet with controlled protein intake and improve the nutritional status of CKD patients.

An interesting role in sarcopenia could be played by n-3 polyunsaturated fatty acid (PUFA) supplementation. PUFAs are known for their protective effects against cardiovascular and inflammatory diseases, but recent studies have also shown that they are likely to attenuate anabolic muscle resistance and may be useful as a therapeutic agent in the treatment of sarcopenia [63]. In line with this hypothesis, Smith et al. have shown that dietary omega-3 fatty acid supplementation increases muscle anabolic signaling activity and insulin/amino acid-mediated increases in muscle protein synthesis in elderly healthy subjects [64]. Few studies have been conducted on patients with CKD, and all the data available are from hemodialysis patients. All studies have shown positive results, highlighting an improvement in the systemic inflammatory state, nutritional and glycemic status, reducing the risk of malnutrition and, in particular, CV events and mortality [63,65].

In the past, the restriction of potassium, sodium, and phosphorus was mandatory. This recommendation led to a reduction in foods rich in these minerals, i.e., fruits, vegetables, whole grains, legumes, and nuts; the restriction of these nutrient-rich foods posed a potential risk because it indirectly promoted the consumption of highly processed foods. These highly processed foods are typically lower in fiber, antioxidants, vitamins, and prebiotics, which can lead to an imbalance in the gut microbiota (dysbiosis) and increased production of uremic toxins, as well as increased production of ammonia compounds and other undesirable effects [66].

The Academy of Nutrition and Dietetics and the National Kidney Foundation have collaborated on an update of the clinical practice guidelines (CPGs) for nutrition in chronic kidney disease (CKD). The new guidelines allow for adjustments in dietary intake to maintain correct serum levels of these minerals and electrolytes, including these healthy foods, and promote an individualized nutritional approach. The new renal nutrition guidelines also make no recommendations regarding the type of protein used in low-protein diets [66,67].

In fact, recent studies have found that not only the amount of protein but also adherence to higher levels of a plant-based diet (the term includes vegetarian, vegan, and Mediterranean diets) in older men with stage 3–5 chronic kidney disease (CKD) is associated with improved insulin sensitivity and lower levels of inflammatory markers [68]. In 2020, Gonzalez–Ortiz et al. conducted a cross-sectional study involving 418 men aged 70–71 years on cystatin-C therapy with an estimated eGFR < 60 mL/min. Data from 7-day food records were used to assess adherence to a plant-based dietary index (PBDi), which assigns positive scores to the consumption of plant-based foods and negative scores to the consumption of animal-based foods. Insulin sensitivity and glucose disposal rate were then assessed. Inflammation was analyzed by serum concentrations of C-reactive protein (CRP) and interleukin (IL)-6. Adherence to a PBDi was moderately associated with higher insulin sensitivity and lower systemic inflammation, suggesting a potential role for these diets in the prevention of metabolic complications in CKD [69].

It is important to ask the following question: “What are the benefits of choosing a plant-based diet to prevent sarcopenia?”. Animal proteins are known to be rich in sulfur-containing amino acids such as methionine and cysteine. These amino acids contribute to an increased acid load in the body and can lead to the production of potentially toxic intermediates such as homocysteine and SAH (S-adenosylhomocysteine). In contrast, plant protein sources tend to be lower in methionine and have a naturally alkaline effect on the body. Plant foods contain bases, such as citrate, which can help reduce metabolic acidosis and slow the progression of chronic kidney disease (CKD). Reducing the acid load in the diet allows the kidneys to excrete more uric acid, resulting in lower serum uric acid levels. Even mild acidosis can cause accelerated muscle wasting, osteoporosis, and hypoalbuminemia, especially if bicarbonate levels are low [57,58].

However, plant proteins are less anabolic than animal proteins; in particular, the essential amino acid (mainly methionine and lysine) content of plant proteins is lower than that of animal proteins, and plant proteins are less digestible than animal proteins [57]. Several studies have been conducted to investigate the muscle response to the long-term effects of plant protein consumption. The results showed that a higher protein intake has favorable effects on muscle mass and function in elderly subjects and that plant protein sources, at low-protein intakes (≈0.8 g/kg/day), have a lower ability to stimulate protein synthesis in muscle and cause muscle mass gain compared to animal proteins. However, the gap between the anabolic effects of plant and animal proteins can be bridged by an adequate (1.1–1.2 g/kg) intake of plant protein [57,70,71]. Furthermore, it has been shown that it is important to consume different plant protein sources with complementary essential AA compositions to optimize a complete AA intake, especially in the elderly [72]. See Table 1 for reviewed studies.

Several strategies have been implemented to enhance the anabolic response induced by plant proteins [57], including:

- Formulation of diets containing different plant protein sources to provide a high-quality AA profile. This approach has also been used successfully in middle-aged patients with CKD, but the nutritional benefits of these combinations have not been investigated in older CKD patients [44].
- Consuming higher amounts of plant protein. This alternative may be attractive to people with CKD who find it difficult to adhere to a low-protein diet (LPD) or who experience some degree of muscle wasting when following a plant-based LPD.
- Combining plant and animal proteins.
- Supplementing plant-based LPDs with essential AA or KA. A vegetarian diet combined with a low-protein intake and amino acid/keto acid (AA/KA) supplementation appears to be a viable option for people with chronic kidney disease (CKD) and may provide adequate nutrition for those who adhere to this treatment. However, further research is needed to determine the effectiveness of these dietary strategies on postprandial muscle protein synthesis.
- Increasing plant sources to obtain high-quality AA profiles.

## 5. Conclusions

In conclusion, loss of muscle mass is a common complication in patients with chronic kidney disease (CKD). This loss of muscle mass is caused by increased protein catabolism and decreased protein synthesis, resulting in a negative protein balance.

Nutritional strategies play a crucial role in preventing and improving muscle loss and sarcopenia in CKD patients. Protein restriction is often used to manage uremic toxicity and slow the progression of renal failure but should be carefully balanced with adequate energy intake to maintain a neutral or positive nitrogen balance. Low-protein diets (LPDs) and very low-protein diets (VLPDs) supplemented with essential amino acids and ketoacids have shown beneficial effects in preserving muscle mass and slowing the progression of CKD. LPDs can reduce muscle protein breakdown and improve the efficiency of protein metabolism, while VLPDs can further reduce the negative net protein balance and improve muscle protein turnover.

The protein requirements of patients with CKD, particularly the elderly, are complex and may differ due to age-related factors, reduced renal function, changes in body composition, and other considerations. Elderly patients with CKD may require higher protein intake to maintain lean body mass and prevent sarcopenia. However, the optimal protein intake for elderly CKD patients is still controversial, and individualized approaches are needed.

Overall, nutritional interventions, including protein restriction with adequate energy intake and supplementation with essential amino acids and ketoacids, may be effective in preserving muscle mass and improving muscle strength in pre-dialysis CKD patients. However, further research is needed to better understand the optimal protein intake and nutritional strategies for different stages of CKD and specific patient populations.

## Figures and Tables

**Table 1:** Summary of principal studies and paper evaluating the impact of the diet on sarcopenia in CKD patients.

References	Study Features	Subjects	Aim of the Study	Intervention	Observed Effects	Conclusions
Rhee et al., (2018) [45]	Meta-analysis: Randomized, self-controlled, parallel, and cross-over trials. Included 16 controlled trials of a low-protein diet in CKD, each with at least 30 participants.	CKD patients Exclusion criteria: ESRD patients and those receiving dialysis treatment	Evidence of the impact of a low-protein diet in the management of uremia and its complications in patients with CKD.	Studies were divided into groups: - Those comparing LPD (0.4–0.8 mg/kg/day) with higher-protein diets (>0.8 g/kg/day); - Those comparing VLPD (<0.4 g/kg/day) with LPD; - Those involving other comparisons.	Low-protein diet vs. higher-protein diets: - ↓ risk of progression to ESRD in LPD patients (ss); - ↑ serum bicarbonate in LPD patients at 1 year (ss); - Serum phosphorus was comparable at 1 year (ss); - No data on PTH and calcium. Very low-protein diet vs. low-protein diet: - ↓ risk of progression to ESRD in VLPD patients (ss); - ↑ GFR in VLPD patients at 1 year (ss); - <GFR decline in VLPD (nss); - <serum urea at 1 year in VLPD (nss); - No data on bicarbonate, phosphorus, PTH, and calcium; - None of the studied ↑ risk of protein-energy wasting or cachexia.	A low-protein diet: - Conservative management of non-dialysis-dependent CKD; - It is considered a potential option for CKD patients to avoid or defer dialysis initiation and to slow down the progression of CKD; - The risk of protein-energy wasting and cachexia remains minimal.
Barsotti et al., (1981) [53]	RCT study	56 CKD patients	Evaluate the effects on renal function of an LPD supplemented with essential amino acids and KAs and of hemodialysis and free protein supply in CKD patients.	1° group: *n* = 31 cases: conventional LPD; 2° group: *n* = 12 cases: VLPD supplemented with essential amino acids and Kas; 3° group: *n* = 13 cases: no dietary intervention.	In the 1° group: ↓ creatinine clearance linearly with time; In the 2° group: creatinine clearance remained constant with only one exception in which it continued to decline; In the 3° group: the deterioration of creatinine clearance was markedly accelerated.	LPD supplemented with KAs and amino acids allowed a slowing of the progression of renal disease and reduced CV risk.
Zemchenkov et al., (2016) [60]	RCT Study	Intervention group: *n* = 96 CKD patients stage 3B-5; Control group: *n* = 96.	Evaluate the effect of LPD supplemented with EAAs/KAs therapy on the rate of eGFR decline in CKD patients stage 3B-5.	LPD (0.60 g protein/kg body weight/day—30 kcal/kg/day of energy) + EAA/KA (prescribed dose—one pill per 5 kg body weight)	In the treatment group: ss ↓ of eGFR rate.	The study showed a reduction in CKD progression, particularly in older patients with higher time-averaged proteinuria and lower phosphate levels, particularly in women.
Milovanova et al., (2018) [61]	RCT Study	79 CKD 3b-4 patients	Compare the possibilities of LPD + KA and isolated LPD in their impact on FGF-23 in patients with CKD stage 3b to 4.	1° group: *n* = 42 patients: LPD + KA; 2° group: *n* = 37 patients: LPD.	In the 1° group: ss ↓ BMI and muscle body mass; In the 2° group: ss ↓ FGF-23.	PD + KA provides support for nutrition status and contributes to more efficient correction of FGF-23. A prolonged LPD alone may lead to malnutrition.
Wu et al., (2013) [62]	RCT Study	109 CKD 3–4 patients Intervention group: *n*= 55; Control group: *n* = 54.	Evaluate the effects of an NPC supplement on renal function and nutritional status in patients on a low-protein diet.	All groups: daily protein intake of 0.6 to 0.8 g and a daily energy intake of 30 to 35 kcal/kg; Intervention group: + 200-kcal NPC supplement daily.	In the intervention group: - ss ↓ in dietary protein intake and urine protein excretion levels; - ss ↓ in serum levels of creatinine and urea nitrogen; - ss ↑ eGFR. No significant differences were observed in the control group.	The NPC supplement improved patient adherence to the low-protein diet and reduced urine protein excretion in patients with CKD.
Gonzalez-Ortiz et al., (2020) [69]	Cross-sectional Study	418 CKD stage 3–5 patients	Explore associations between adherence to plant-based diets and measures of insulin sensitivity and inflammation in men with CKD stage 3–5.	Information from 7-day food records was used to evaluate the adherence to a PBDi, which scores the intake of plant foods positively and animal foods negatively.	A higher PBDi score remained associated with higher glucose disposal rate and insulin sensitivity, as well as with lower levels of IL-6 and CRP.	Adherence to a plant-based diet was associated with higher insulin sensitivity and lower inflammation, supporting a possible role of plant-based diets in the prevention of metabolic complications of CKD.

CKD: chronic kidney disease; RCT: randomized controlled study; LPD: low-protein diet; VLPD: very low-protein diet; ESRD: end-stage renal disease; PTH: parathyroid hormone; nss: not statistically significant; KAs: ketoanalogues; CV: cardiovascular; eGFR: glomerular filtration rate; EAA: essential amino acids; BMI: body mass index; NPC: non-protein calorie; ss: statistically significant; IL: inflammatory cytokines; CRP: c-reactive protein; PBDi: plant-based diet index.

## Data Availability

Not applicable.
